# ESRP1 drives epithelial-mesenchymal transition by activating EPAC-RAP1A signaling axis

**DOI:** 10.3389/fmed.2026.1734619

**Published:** 2026-02-06

**Authors:** Ruixin Qi, Jiaqi Wang, Guocang Cheng, Tingting Zhao, Zhengyu Hu, Jialin Yu, Yuanyuan Jia, Juan Chen

**Affiliations:** 1 Ningxia Key Laboratory of Clinical and Pathogenic Microorganisms, Institute of Medical Science, General Hospital of Ningxia Medical University, Yinchuan, China; 2 Department of Respiratory and Critical Care Medicine, Qingyang People’s Hospital, Qingyang, Gansu, China; 3 Department of Respiratory and Critical Care Medicine, General Hospital of Ningxia Medical University, Yinchuan, China

**Keywords:** Epac-Rap1a signaling, epithelial, epithelial splicing regulatory protein 1 (ESRP1), epithelial-mesenchymal transition (EMT), idiopathic pulmonary fibrosis, single-cell RNA sequencing (scRNA-seq)

## Abstract

**Background:**

Idiopathic pulmonary fibrosis (IPF) is a fatal lung disease characterized by epithelial-mesenchymal transition (EMT) as a key pathological feature. The molecular mechanism of EMT is not fully understood. Hence, the current study aimed to investigate the pathogenesis of EMT, which focus on the function of Epithelial Splicing Regulatory Protein 1 (ESRP1) in regulating EMT.

**Methods:**

The present study utilized bleomycin (BLM) to establish mouse models of IPF. Then, single-cell RNA sequencing (scRNA-seq) of entire lung tissue was employed to delineate transcriptional alterations in epithelial cells and to nominate prospective regulators of EMT. The target gene was subsequently validated *in vivo* and *in vitro* by qPCR, western blot, and immunofluorescence. Furthermore, an EMT model was established in TGF-β1–treated MLE-12 alveolar epithelial cells. Lentivirus or siRNA was hired to modulate the expression of target gene and elucidate its mechanistic contribution to EMT.

**Results:**

ScRNA-seq revealed marked up-regulation of the ESRP1 in alveolar epithelial cells compared with PBS-treated controls. Subsequent mechanistic interrogation in primary and MLE-12 alveolar epithelial cells demonstrated that knockdown of ESRP1 suppressed, whereas its overexpression potentiated, the expression of Epac, Rap1a, and N-cad which were key effectors of EMT. Importantly, Co-IP (Co-Immunoprecipitation) showed that there was interaction between ESRP1, Epac, and Rap1a. Silencing of either Epac or Rap1a did not reciprocally alter ESRP1 expression, confirming an upstream regulatory hierarchy.

**Conclusion:**

Our findings demonstrate that ESRP1 upregulation in alveolar epithelial cells drives IPF progression by promoting EMT via the Epac–Rap1a axis.

## 1 Introduction

Idiopathic pulmonary fibrosis (IPF), a progressive and fatal interstitial lung disease, manifests through fibroblast hyperproliferation, pathological extracellular matrix (ECM) deposition, and irreversible lung function decline (1, 2). Despite FDA-approved therapies (pirfenidone/nintedanib), clinical outcomes remain suboptimal with marginal survival benefits (3–5), underscoring an urgent need to elucidate molecular drivers of fibrosis, particularly epithelial-mesenchymal transition (EMT), a cornerstone of IPF pathogenesis (6, 7). In IPF, alveolar epithelial injury triggers TGF-β1-dominated EMT, transforming epithelial cells into collagen-secreting myofibroblasts via Smad/MAPK pathways (8, 9). This process is defined by E-cadherin (E-cad)/ZO-1(Zonula Occludens-1) suppression and α-SMA (alpha-Smooth Muscle Actin)/Vimentin (Vim) induction (10–12), yet upstream regulators orchestrating EMT initiation remain poorly defined.

In this research, we established murine IPF model, and single-cell RNA sequencing revealed marked up-regulation of ESRP1 in epithelial cells. According to previous study, ESRP1, an RNA-binding protein restricted to epithelial lineages, orchestrates the alternative splicing of multiple transcripts that regulate cell–cell adhesion, motile behavior, and intracellular signaling cascades (13, 14). While ESRP1’s tumor-suppressive or oncogenic roles has been well-documented (15–21), its function in IPF is poorly characterized. Growing evidence demonstrated that ESRP1 orchestrates the alternative splicing of multiple transcripts, including bFGF (basic Fibroblast Growth Factor), FGFR2 (Fibroblast Growth Factor Receptor 2), CD44 (Cluster of Differentiation 44), CTNND1 (Catenin Delta 1/p120-catenin) and ENAH (Enabled Homolog), thereby modulating epithelial plasticity and migratory capacity (22–27). A recent study also implicates an ESRP1-mediated circRNA network in TGF-β1-driven EMT in breast cancer (28).

Hence, the current study aimed to investigate the roles of ESRP1 in IPF-associated EMT. We first established mouse model of Bleomycin (BLM)-induced IPF and subjected to scRNA-seq. ESRP1 exhibited the most pronounced up-regulation within injured alveolar epithelial cells. Both experimental models and clinical investigations have shown that ESRP1 exhibits elevated expression in the lungs of individuals with IPF as well as in BLM-treated mouse lungs. Furthermore, spatial immunoprofiling revealed co-induction of ESRP1 with Epac and Rap1a across fibrotic lungs. In MLE-12 cells, knockdown or overexpression of *esrp1* elicited concordant changes in Epac (Exchange Protein directly Activated by cAMP), Rap1a (Ras-related Protein 1a), and N-cad (N-cadherin), whereas silencing of *epac* or *rap1a* left ESRP1 expression unaltered. Co-IP showed that there was interaction between ESRP1, Epac and Rap1a. To date, ESRP1 acts as an upstream gatekeeper that licenses EMT via the Epac–Rap1a axis and represents a tractable therapeutic target for EMT-driven fibrosis in IPF.

## 2 Results

### 2.1 Single cell sequencing showed that ESRP1 was highly expressed in epithelial cells of BLM-induced lung fibrosis mouse model

At the start of the experiment (day 0), male C57BL/6J mice aged 6–8 weeks received one intratracheal instillation of BLM at 2 mg/kg, prepared in 50 μL sterile saline. Mice were then monitored for 21 days under SPF housing conditions and euthanized on day 21 for tissue collection and subsequent analyses. After 21 days of BLM treatment, BLM exposure resulted in evident histopathological alterations, as shown by H&E staining, including inflammatory cell infiltration, alveolar enlargement, and septal thickening. Extensive collagen deposition was observed in the tissue sections when examined by Masson’s trichrome staining, primarily localized around bronchioles and within the alveolar interstitium. IHC (Immunohistochemistry) further confirmed elevated expression of Fn1(Fibronectin) (*P* < 0.001), Vim (*P* < 0.001), and α-SMA (*P* < 0.001), indicating enhanced fibrotic activity in BLM-treated lungs (Supplementary Figure 1a). The impact of BLM and normal saline on mouse body weight was monitored over time. In the BLM-treated group, weight gain ceased by day 14, followed by a more pronounced reduction in body weight observed on day 21, indicating progressive systemic effects associated with fibrosis induction (Supplementary Figure 1b). Hydroxyproline assays of lung homogenates demonstrated significantly higher collagen content in the BLM-treated group compared to controls, supporting increased extracellular matrix deposition (Supplementary Figure 1c). Western blot and qPCR analyses showed significant upregulation of fibrosis-related markers, including Fn1, N-cad, α-SMA, and Zeb1 (Zinc Finger E-box Binding Homeobox 1), in lungs from bleomycin-treated mice, relative to controls, at the gene and protein expression levels (*P* < 0.05) (Supplementary Figure 1d). Our data confirm that BLM-treatment reliably produced a model of pulmonary fibrosis, distinguished by extensive inflammatory infiltration and tissue injury. While ESRP1 is well-known for regulating FGFR2 alternative splicing, which we confirmed via qPCR (Supplementary Figure 1e). This alteration did not fully account for the observed fibrotic phenotype. The primers used for this study are summarized in Table 1. Further mechanistic exploration identified the Epac-Rap1a pathway as the predominant mediator through which ESRP1 exerts its pro-EMT effects in this specific context.

**TABLE 1:** *FGFR2-IIIb* and *FGFR2-IIIc* sequence used in this study (Sangon Biotech).

Gene name	Sense	AntiSense
*FGFR2-IIIb*	CAACACCGAGAAGAT GGAGAAG	CACCATGCAGGCGATTAA GA
*FGFR2-IIIc*	CCAGCACTGGAGCCTT ATTAT	GATCCTCTGGCAACTCAT ACTC

*FGFR2-IIIb* and *FGFR2-IIIc*, alternative splicing isoforms of Fibroblast Growth Factor Receptor 2.

**FIGURE 1:**
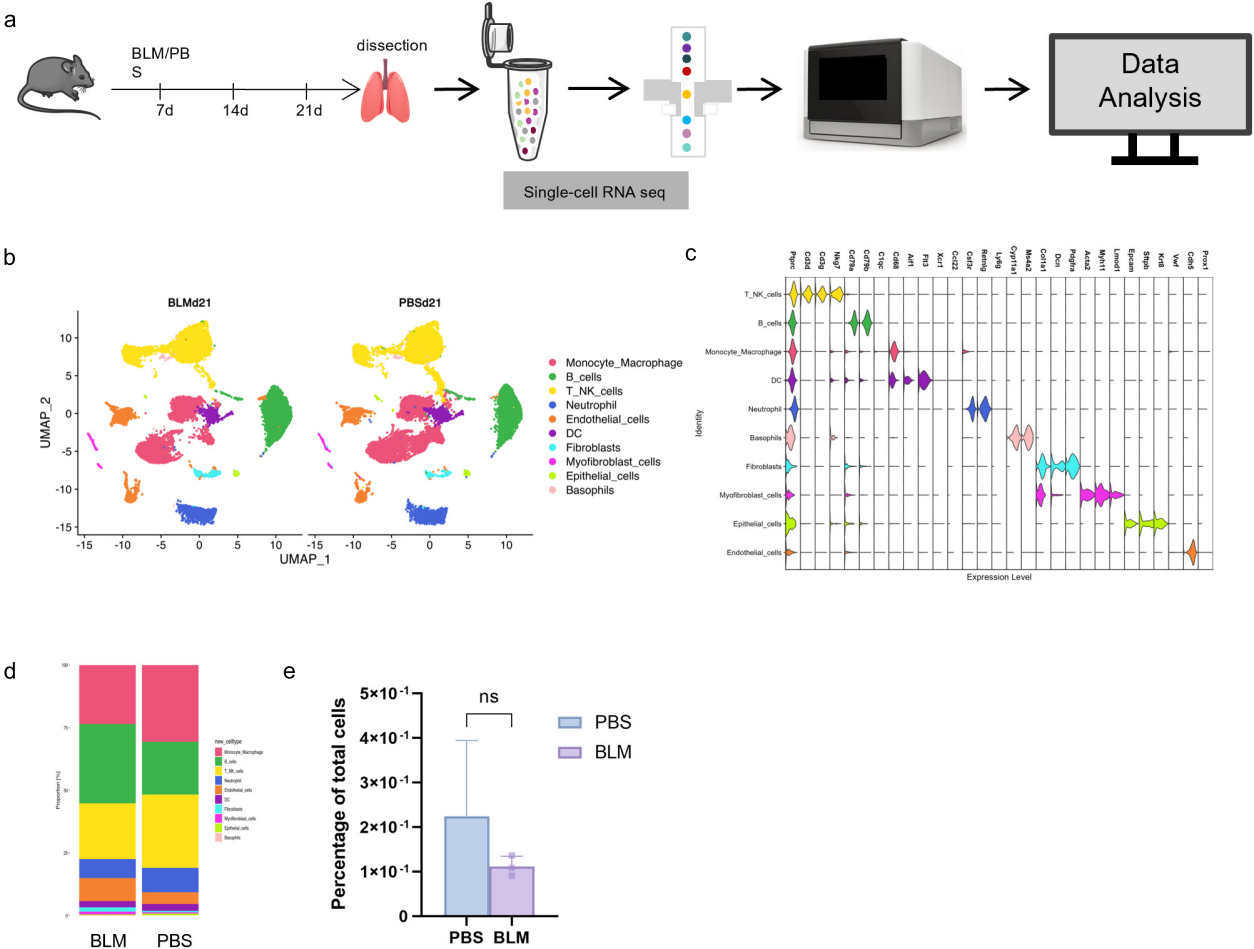
Single-cell landscape of cellular composition in bleomycin-induced pulmonary fibrosis. **(a)** Flow chart of single cell sequencing. **(b)** UMAP plots showing the distribution of lung cell populations from BLMd21 and PBSd21 mice. A total of 10 major cell types were identified and annotated based on canonical markers: Monocyte_Macrophage (red), B cells (green), T/NK cells (yellow), Neutrophils (blue), Endothelial cells (orange), Dendritic cells (DC, pink), Fibroblasts (cyan), Myofibroblast-like cells (purple), Epithelial cells (light green), and Basophils (brown). Color indicates cluster identity. **(c)** Violin plots illustrate the expression profiles of representative marker genes across the annotated cell populations. The *X*-axis represents the top genes for each cell subgroup, while the *Y*-axis denotes the different cell types. **(d)** Bar plot showing the proportional distribution of each cell type between BLM and PBS groups. Notably, fibrotic lungs displayed increased fibroblast and myofibroblast-like populations, with a concomitant reduction in epithelial cells. **(e)** Quantification of epithelial cell proportion (percentage of total cells) showed a decreasing trend in the BLM group compared to PBS, though without statistical significance (ns, unpaired *t*-test).

Furthermore, to explore the cellular heterogeneity in the BLM-induced IPF model, we performed sc-RNA seq of lung tissues collected on day 21 post-treatment from both BLM and PBS groups (Figure 1a). After quality control, we profiled 91,418 cells from total lung samples obtained from 3 PBS control mice and 3 BLM treatment mice, identified 10 discrete cell types based on distinct markers, cells were visualized using UMAP (Uniform Manifold Approximation and Projection) projection and grouped into 10 major cell populations based on canonical marker genes (Figure 1b). The identified clusters included: Monocyte_Macrophage, B cells, T/NK cells, Neutrophils, Dendritic Cells (DCs), Fibroblasts, Myofibroblast-like cells, Epithelial cells, Endothelial cells, and Basophils.

Top marker genes for each cell type were identified via differential expression analysis and visualized in violin plots (Figure 1c). For example, Col1a1 (Collagen Type I Alpha 1) and Acta2 (Actin Alpha 2, Smooth Muscle) in myofibroblasts, Epcam in epithelial cells, and Pecam1 in endothelial cells, confirming robust cell-type annotation.

Comparative analysis of cellular composition between BLM and PBS (Phosphate-Buffered Saline) groups revealed noticeable shifts (Figure 1d). BLM-treated lungs exhibited an increase in fibroblasts and myofibroblast-like cells, accompanied by a reduction in epithelial cells and endothelial cells, suggesting fibrotic remodeling and epithelial injury. Quantification of epithelial cell proportions (Figure 1e) showed a marked decrease in the BLM group compared to PBS, though the difference did not reach statistical significance (ns, *P* > 0.05).

These data demonstrate that bleomycin exposure induces pronounced cellular reorganization in the lung, with expansion of profibrotic mesenchymal populations and concomitant depletion of epithelial compartments, consistent with pathological hallmarks of IPF.

**FIGURE 2:**
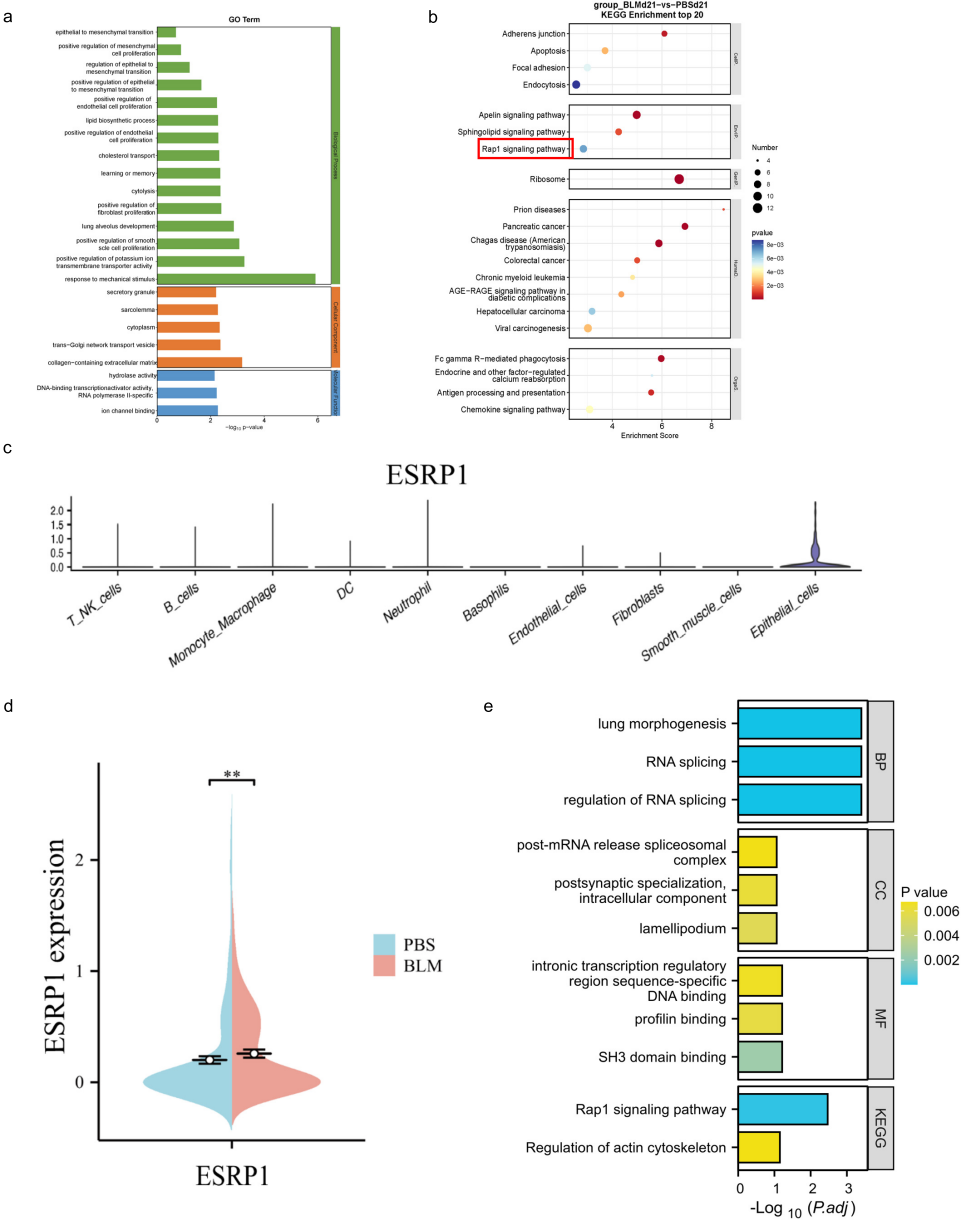
Differential gene expression and functional enrichment of lung epithelial cells in bleomycin-induced pulmonary fibrosis. **(a)** GO analysis of epithelial cell function. **(b)** KEGG pathway analysis of all cells. **(c)** To detect the expression and distribution of ESRP1 in each cell using Loupe software. **(d)** To detect the difference of ESRP1 in epithelial cells of lung tissue between two groups of mice. **(e)** Function and pathway analysis of ESRP1 gene with GO and KEGG.

Next, we utilized GO (Gene Ontology) and KEGG (Kyoto Encyclopedia of Genes and Genomes) pathway analyses were employed to investigate the functional roles of genes highly expressed in epithelial cells. The GO functions of 30 TOP genes screened by single-cell sequencing showed significant changes in the functions of positive regulation of EMT and response to mchanical stimulus (Figure 2a). Meanwhile, based on functional annotations, it was found that the esrp1 was involved. The KEGG enrichment analysis demonstrated that epithelial cell–associated genes were distributed across the RAP (Ras-related Protein), apelin, and sphingolipid signaling pathways (Figure 2b). These results suggested that epithelial cells are critical population of EMT.

Subsequent genetic screening revealed specific expression of esrp1 in epithelial cells (Figure 2c), and significant elevation in BLM-induced mice compared to control group (Figure 2d). To further elucidate the function of ESRP1, GO and KEGG pathway analysis were conducted. The results showed that ESRP1 regulates RNA splicing and plays a key role in modulating the Rap1 signaling pathway and actin cytoskeleton, processes crucial for fibrosis progression (Figure 2e).

### 2.2 ESRP1, Epac, and Rap1a are up-regulated in BLM-induced pulmonary fibrosis and IPF patients

**FIGURE 3:**
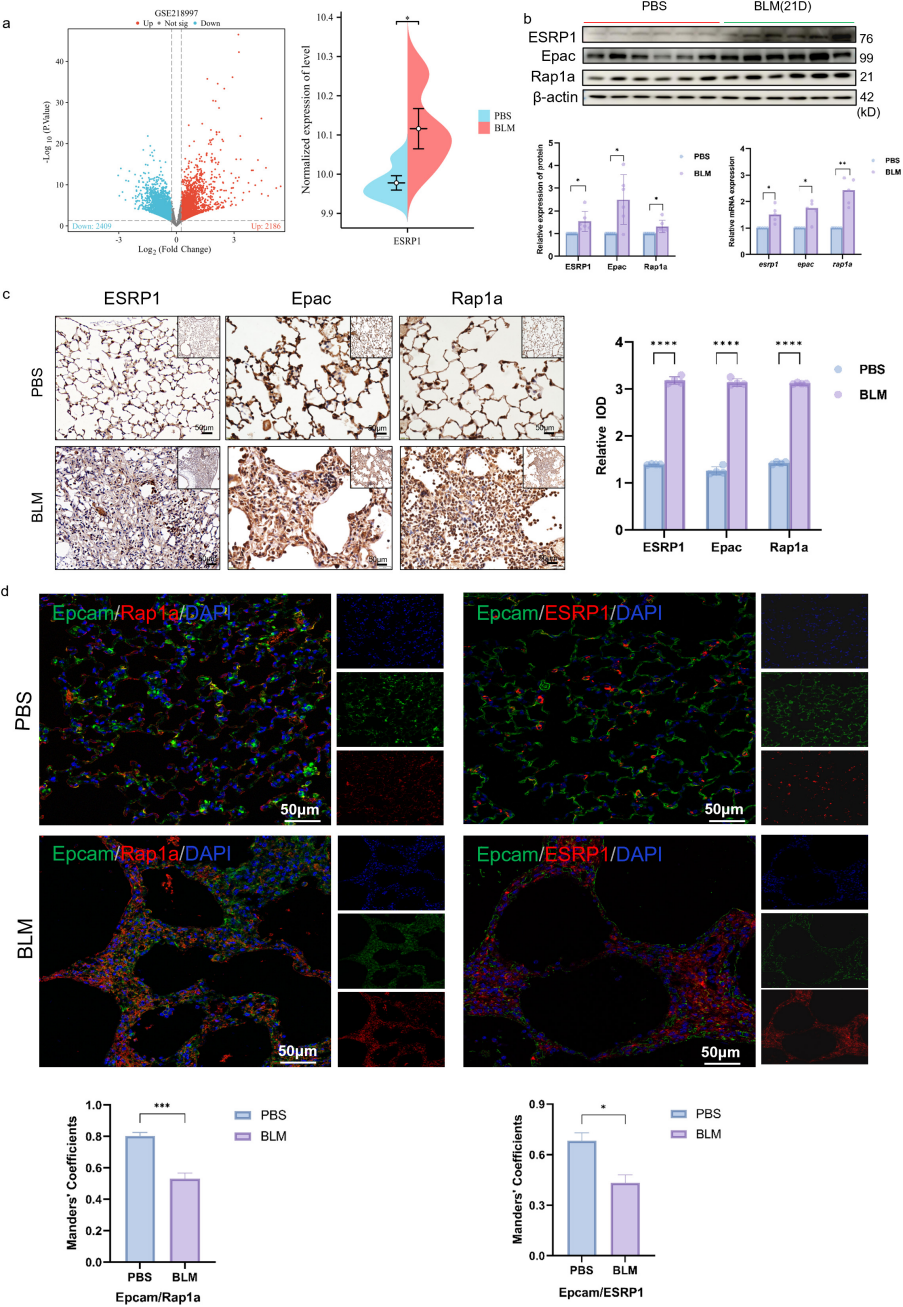
ESRP1 and Rap1a were both upregulated in the lung tissues of BLM-induced pulmonary fibrosis models. **(a)** GSE218997 data set search found that ESRP1 increased in BLM-induced lung tissue of mice. **(b)** In mouse lungs, ESRP1, Epac, and Rap1a levels were analyzed by western blot after 3 weeks of bleomycin or saline administration. **(c)** IHC was used to examine the levels of ESRP1, Epac, and Rap1a in the lung tissues of BLM-induced models (40 × ). **(d)** Multiplex immunofluorescence showing expression and co-localization of Epcam with ESRP1 or Rap1a in lung sections from indicated mouse groups (scale bar, 50 μm). Manders’ correlation coefficients quantify the degree of co-localization. Statistically significant differences are indicated as *P < 0.05, **P < 0.01, ***P < 0.001, and ****P < 0.0001.

To further clarify the expression of ESRP1, the current study analyzed the expression changes of ESRP1 in the public database GSE218997. The results showed that ESRP1 was highly expressed in BLM-induced lung tissue of mice (Figure 3a). In addition, KEGG enrichment analysis indicated a significant association between the ESRP1 and Rap1a signaling pathways.

Small GTPases serve as multifunctional regulators involved in numerous cellular activities (29). As guanine nucleotide exchange factors (GEFs), Epac proteins mediate the activation of Rap small GTPases. Two isoforms are present in mammals: Epac1, expressed broadly across tissues, and Epac2 (RapGEF4 in humans), which shows tissue-restricted expression in the brain, kidney, and pancreas (30–32). Rap1a activation can facilitate EMT by modulating the expression of cell adhesion molecules and orchestrating cytoskeletal reconfiguration, thereby influencing cell shape and movement (33, 34). Pathological activation of the Epac–Rap1a axis in fibrotic tissues contributes to the amplification of EMT processes, subsequently enhancing extracellular matrix production and deposition, thereby aggravating the extent of fibrosis within lung tissue (35).

Hence, this study utilized western blot analysis together with qPCR to assess the expression patterns of the Epac–Rap1a signaling axis. The results validated the increased expression of ESRP1, Epac, and Rap1a among mice exposed to BLM when evaluated against the control group (Figure 3b). IHC was further conducted to confirm this result. As shown in Figure 3c, the proteins which indicated by brown dots were located within alveolar and bronchial epithelial cells and exhibited higher expression levels in BLM group mice compared to the control group (Figure 3c).

Furthermore, multiplex immunofluorescence revealed a progressive loss of the epithelial marker Epcam in BLM-exposed lungs compared with controls. Concomitantly, both ESRP1 and Rap1a signals were markedly elevated in fibrotic foci on day 21 (Figure 3d). These results demonstrate that ESRP1 contributes to the development in BLM-treated mice, particularly through its impact on epithelial cells.

**FIGURE 4:**
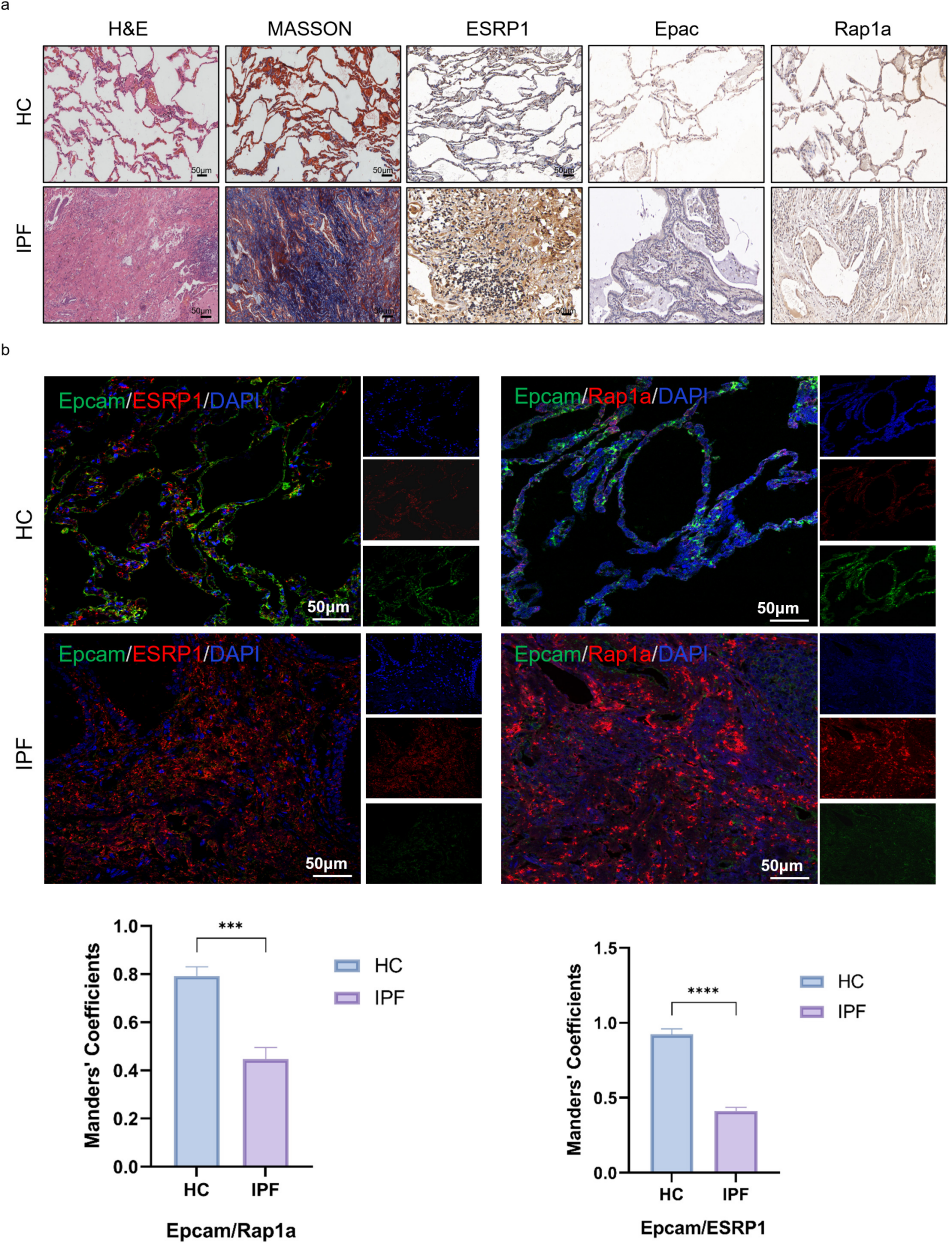
ESRP1 and Rap1a were both upregulated in the lung tissues of IPF patients. **(a)** IPF was evaluated using H&E staining, while collagen deposition through Masson’s staining, and the levels of ESRP1, Epac, and Rap1a was detected immunohistochemically in healthy and IPF patients. **(b)** Multiplex immunofluorescence showing expression and co-localization of Epcam with ESRP1 or Rap1a in both HC and IPF groups (scale bar, 50 μm). Manders’ correlation coefficients quantify the degree of co-localization. Statistically significant differences are indicated as *P < 0.05, **P < 0.01, ***P < 0.001, and ****P < 0.0001.

To validate the expression of ESRP1 in patients with pulmonary fibrosis, lung tissues from both normal and fibrotic samples were collected. H&E staining revealed severe fibrosis in IPF patients compared to healthy controls, characterized by inflammatory cell infiltration and alveolar hemorrhage. Additionally, there was noticeable alveolar enlargement and thickening of the alveolar septa. Masson’s trichrome staining indicated substantial collagen deposition, visible as intense blue staining around the bronchial walls and alveolar septum in IPF patients. Furthermore, IHC analysis demonstrated that protein levels of ESRP1, Epac, and Rap1a were elevated in IPF patients (Figure 4a). IF analysis further corroborated the increased ESRP1 and Rap1a expression in IPF tissues, alongside a reduction in the epithelial cell marker Epcam (Epithelial Cell Adhesion Molecule). Additionally, no significant colocalization was observed between Epacm and ESRP1, or between Epcam and Rap1a (Figure 4b). In healthy lung tissue, alveolar structures remained intact and exhibited only minimal ESRP1 expression, primarily localized to alveolar and bronchial cells. In contrast, lung tissue from IPF displayed disrupted alveolar architecture, proliferation of interstitial fibrous tissue, and elevated Esrp1 expression in bronchial epithelial cells as well as compressed alveolar surfaces. Collectively, these findings indicate that ESRP1 is specifically enriched in epithelial cells residing in the lungs of patients suffering from IPF.

### 2.3 ESRP1 regulates Epac-Rap1a to promote EMT in MLE-12 cells

**FIGURE 5:**
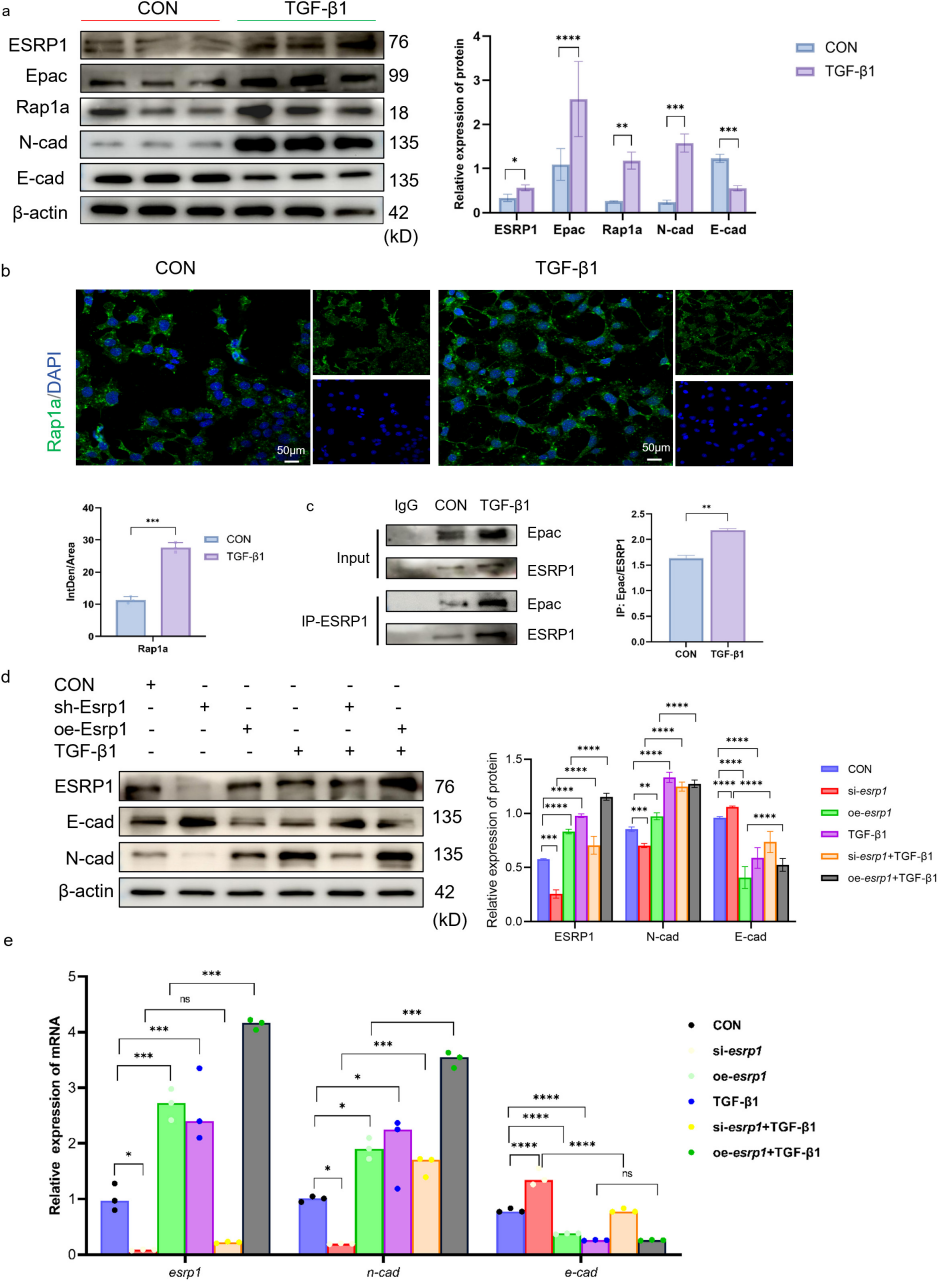
ESRP1 regulates Rap1a to promote EMT in MLE-12 cells. **(a)** The protein expression levels of ESRP1, Epac, Rap1a, N-cad, and E-cad were measured in MLE-12 cells treated with 10 ng/ml TGF-β1 for 24 h. **(b)** IF analysis showed Rap1a (green signal) expression in both Control and TGF-β1-treated groups. **(c)** Co-IP demonstrating the interaction between ESRP1 and Epac in mouse MLE-12 lung epithelial cells. The protein detection results after IP were used for the quantitative analysis of protein-protein interactions. **(d,e)** Following 48 h of lentiviral exposure, MLE-12 cells were subjected to TGF-β1 stimulation for 24 h, siRNA, lentivirus, siRNA+ TGF-β1 and lentivirus + TGF-β1, the levels of ESRP1, N-cad, and E-cad were determined by qPCR and Western Blot. Quantified by densitometric analysis. The data is expressed as average ± SD. Statistically significant differences are indicated as *P < 0.05, **P < 0.01, ***P < 0.001, and ****P < 0.0001.

*In vitro* studies, TGF-β1 serves as a central mediator initiating EMT. Therefore, protein expression analysis was conducted to evaluate EMT markers in 10 ng/mL TGF-β1 stimulated mouse lung epithelial cells (MLE-12). Western blot revealed that TGF-β1 markedly up-regulated ESRP1 protein and simultaneously triggered an EMT signature: E-cad was substantially down-regulated, whereas N-cad expression was significantly elevated (Figure 5a). We also noted enhanced protein expression of Epac and Rap1a in TGF-β1-treated MLE-12 cells compared to the control group (Figure 5a).

Cellular IF (Immunofluorescence) assays further confirmed TGF-β1 treatment increased the expression of Rap1a in the cytoplasm (Figure 5b). KEGG pathway enrichment predicted a potential association between ESRP1 and the Rap1 signaling cascade. To assess the interaction between ESRP1 and Epac, Co-IP was performed on lysates from MLE-12 cells treated with or without TGF-β1. The results showed that anti-ESRP1 antibody co-precipitated Epac from cell lysates. In contrast, normal IgG control antibody failed to precipitate either protein, confirming the specificity of the interaction. Quantitative analyses revealed that TGF-β1 treatment enhanced the interaction between ESRP1 and Epac (36, 37) (mean ± SEM, *n* = 3, ***P < 0.001; Figure 5c). Thus, TGF-β1 stimulation significantly enhanced the Co-IP enrichment of both Epac and ESRP1, indicating a ligand-induced increase in their mutual association. Moreover, TGF-β1 treatment markedly enhanced this interaction, as quantified by densitometric analysis of precipitated proteins.

Furthermore, MLE-12 cells were transduced with lentiviral vectors to silence or overexpress *esrp1*, and its expression were quantified by Western Blot and qPCR. *Esrp1* knockdown markedly reduced the expression of N-cad, whereas E-cad was significantly increased. Conversely, *esrp1* overexpression elevated N-cad transcripts and concurrently suppressed E-cad (Figures 5d,e). These reciprocal changes indicate that ESRP1 transcriptionally regulates EMT.

**FIGURE 6:**
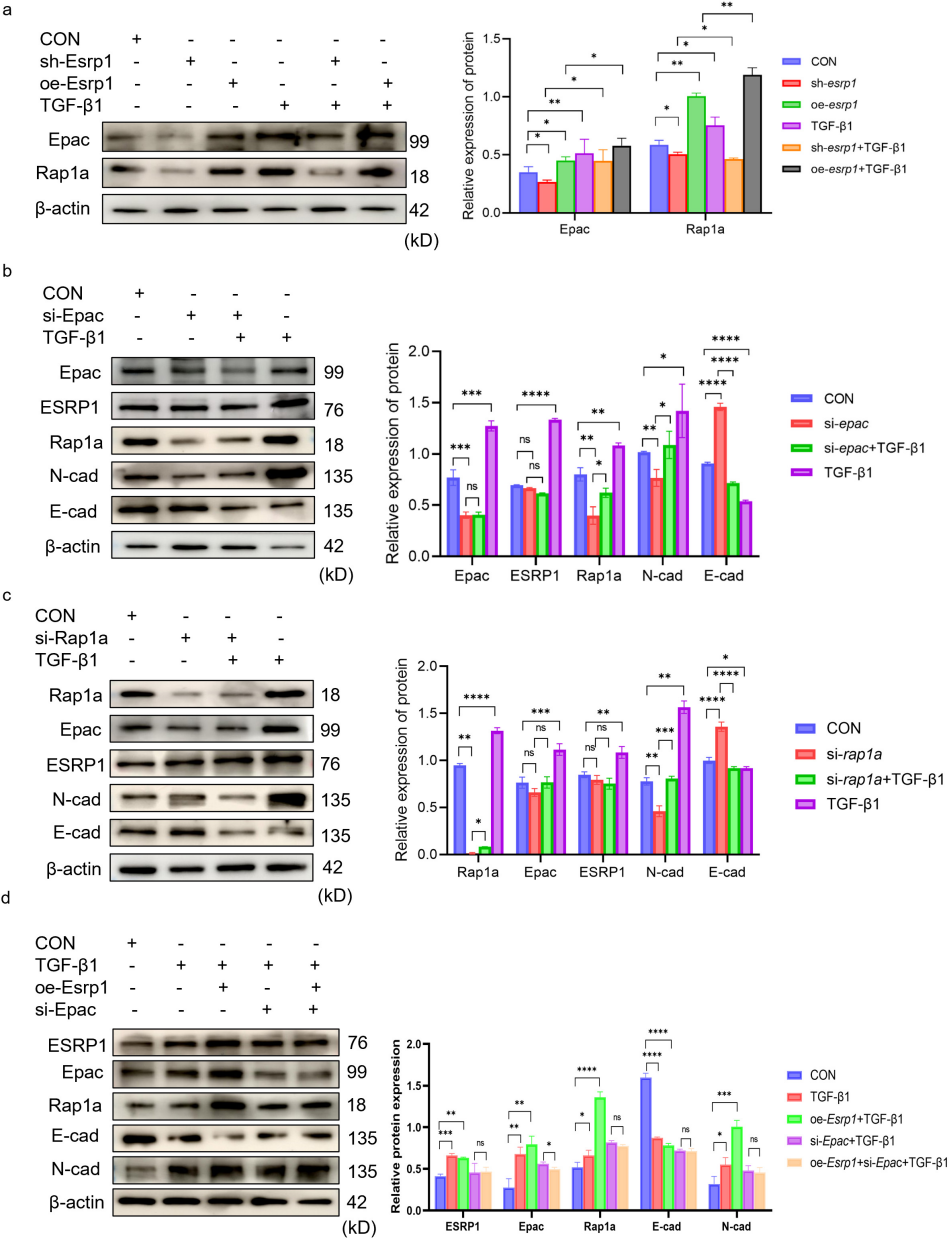
ESRP1 promote EMT via Epac-Rap1a in MLE-12 cells. **(a)** Lentiviral *esrp1* knockdown or overexpression induces parallel changes in Epac and Rap1a. **(b)** siRNA-mediated *epac* silencing does not alter ESRP1 but decreases N-cad and increases E-cad. **(c)** siRNA-mediated *rap1a* silencing leaves Epac and ESRP1 unchanged while similarly reducing N-cad and elevating E-cad. Bar graphs show mean ± SEM; *P < 0.05, **P < 0.01. ***P < 0.001. ****P < 0.0001. **(d)** Representative Western blot and quantitative analysis of Rap1a, Epac, ESRP1, E-cad, and N-cad in cells treated with TGF-β1 or without *esrp1* overexpression and/or *epac* silencing.

To elucidate ESRP1 function, lentivirus-mediated gene perturbation was performed in MLE-12 cells. Compared with scrambled controls, *esrp1* silencing markedly attenuated the expression of Epac, Rap1a and N-cad, while reciprocally enhancing E-cad abundance. Conversely, overexpression *esrp1* elevated Epac, Rap1a and N-cad, accompanied by a concomitant reduction in E-cad (Figure 6a). Collectively, these data demonstrate that ESRP1 positively modulates Epac and Rap1a expression and actively drives EMT.

To clarify the regulatory hierarchy among ESRP1, Epac, and Rap1a, MLE-12 cells were transfected with siRNAs targeting *epac* and *rap1a*. Knockdown of *epac* markedly reduced Rap1a and N-cad levels while increasing E-cad (Figure 6b); conversely, silencing *rap1a* decreased N-cad and elevated E-cad without affecting Epac abundance (Figure 6c). These results corroborate that the Epac–Rap1a axis promotes EMT. Critically, neither Epac nor Rap1a depletion altered ESRP1 expression, whereas *epac* knockdown Rap1a expression. Collectively, the data establish that ESRP1 operates upstream of Epac and Rap1a, with Epac positioned upstream of Rap1a in this signaling cascade. These findings indicated that ESRP1 facilitated TGF-β1-induced EMT by activating Epac-Rap1a signaling *in vitro*.

Upon TGF-β1 stimulation, the protein levels of Rap1a, Epac, ESRP1, and N-cadherin were markedly increased compared with the CON group, whereas E-cadherin was progressively reduced. Notably, ESRP1 overexpression further amplified the TGF-β1–induced changes, as evidenced by higher Rap1a/Epac/ESRP1/N-cadherin expression and a more pronounced decrease in E-cadherin in the oe-Esrp1 + TGF-β1 group. In contrast, in the si-Epac + TGF-β1 group, Rap1a, Epac, ESRP1, and N-cadherin levels were reduced and E-cadherin was elevated relative to the oe-Esrp1 + TGF-β1 group. Importantly, no significant differences were observed in EMT-associated markers or the target proteins between si-Epac + TGF-β1 and si-Epac + oe-Esrp1 + TGF-β1 groups, indicating that Epac knockdown prevented ESRP1 overexpression induce EMT marker alterations under TGF-β1 stimulation (Figure 6d).

## 3 Discussion

Integrated analyses of clinical IPF samples, mouse, cell models, and functional interventions reveal a critical role for the ESRP1-Epac-Rap1a signaling axis in driving EMT in alveolar epithelial cells during IPF. Our results showed that ESRP1 is specifically high expressed in IPF lung tissue and TGF-β_1_-stimulated MLE-12 cells, correlating positively with fibrotic severity. Gain- and loss-of-function experiments demonstrate that ESRP1 directly promotes the expression of Epac and Rap1a, consequently upregulating N-cad and downregulating E-cad. It is worth noting that interference with Epac or Rap1a does not affect ESRP1 expression, positioning ESRP1 upstream within this signaling cascade.

While ESRP1 is a well-established RNA splicing regulator that typically maintains epithelial integrity and suppresses EMT in cancer, our findings reveal its paradoxical pro-fibrotic role in IPF. Within the IPF microenvironment, TGF-β_1_ induces aberrant ESRP1 overexpression, which disrupts epithelial homeostasis to promote fibrosis. We propose that chronic inflammatory signals in fibrotic diseases subvert ESRP1’s physiological function, converting it to a pro-fibrotic effector. Mechanistically, we identify a novel TGF-β_1_/ESRP1- Epac-Rap1a axis driving EMT in IPF. KEGG and experimental validation demonstrate ESRP1’s transcriptional/post-transcriptional control of Rap1a signaling through this axis. Crucially, Epac/Rap1a knockdown phenocopied ESRP1 loss-induced EMT inhibition without altering ESRP1 levels, confirming their essential downstream effector roles. This aligns with conserved profibrotic Rap1a mechanisms (35), suggesting cross-fibrotic disease relevance.

ESRP1 warrants evaluation as a novel biomarker for IPF diagnosis and disease activity monitoring. Furthermore, targeting ESRP1 or its downstream effectors Epac and Rap1a presents a promising therapeutic strategy to inhibit pathogenic EMT while potentially preserving basal epithelial function, offering greater specificity than broad-spectrum antifibrotics. Existing Epac inhibitors (21), primarily explored in oncology, could be repurposed for fibrotic diseases based on this mechanistic foundation.

While several studies indicate that ESRP1’s downregulation is associated with tumor progression and EMT, our findings suggest that ESRP1 overexpression in lung epithelial cells contributes to fibrosis. Pathway analysis using KEGG highlighted the pivotal role of ESRP1 in modulating Rap1a signaling and actin cytoskeletal remodeling. The reduction in epithelial cells observed in this study may be due to increased ESRP1 expression, which promotes their transition to fibroblasts via the Epac-Rap1a pathway. We further investigated whether the impact of ESRP1 on EMT is mediated through the Epac-Rap1a pathway. Our results demonstrated that Epac knockdown effectively neutralized the regulatory influence of ESRP1 on TGF-β1 induced EMT markers. Even with the upregulation of ESRP1, cells with depleted Epac remained unresponsive in terms of EMT marker expression, suggesting that ESRP1-mediated EMT regulation is Epac-dependent. This identifies the ESRP1-Epac-Rap1a axis as a critical molecular link in controlling cellular identity and mesenchymal transformation.

In spite of the strength of the evidence presented, several limitations of the current work should be recognized. First, the absence of human-derived epithelial cells in our *in vitro* experiments restricts the direct applicability of these findings to clinical settings. The heterogeneity in human IPF and potential differences in EMT pathways across species suggest that further studies using human lung tissue or primary epithelial cells from IPF patients would provide a more accurate picture of ESRP1’s role in EMT. Furthermore, although we identified Epac-Rap1a as a downstream signaling pathway of ESRP1, other pathways may also contribute to EMT in IPF and should be explored in future research.

Our study underscores the role of Esrp1 in promoting EMT and fibrosis via Epac-Rap1a signaling, reinforcing the importance of EMT in IPF progression. Our findings propose ESRP1 as a potential therapeutic target, although further research is required to elucidate its mechanism and evaluate its clinical relevance in human patients. By addressing these limitations and further investigating ESRP1’s multifaceted role, future research may yield novel insights and therapeutic avenues for combating IPF.

## 4 Conclusion

In summary, our study demonstrated that ESRP1 is significantly upregulated in alveolar epithelial cells during the progression of IPF. Furthermore, ESRP1 facilitated EMT by activating Epac-Rap1a axis, ultimately leading to the advancement of IPF. By demonstrating the role of ESRP1 in EMT, this study provides a theoretical foundation for exploiting ESRP1 as a therapeutic target in the design of new treatment strategies for pulmonary fibrosis.

## 5 Materials and methods

### 5.1 Lung tissue collection

Written informed consent was secured from every participant prior to enrollment, and the study protocol was reviewed and approved by the Institutional Review Board at the General Hospital of Ningxia Medical University (Approval No. KYLL-2023-0516) and conducted in accordance with NIH guidelines. Patients diagnosed with lung cancer and lung fibrosis, as identified by CT scans, were recruited from Ningxia, Yinchuan. Samples were collected from areas ≥ 5 cm away from the tumor as healthy controls. The tissue collection occurred between January 2024 and October 2024. IPF diagnosis was based on clinical history, physical exams, pulmonary function tests, HRCT (High-Resolution Computed Tomography), and confirmed by lung biopsy, in alignment with the recommendations issued jointly by the American Thoracic Society (ATS) and the European Respiratory Society (ERS) (3). Healthy Control (HC) samples were obtained from lung cancer patients with histologically normal lung tissue. Additionally, lungs from healthy organ donors and IPF transplant donors were used. All tissue usage was approved by the relevant institutional review boards.

### 5.2 Reagents and antibodies

Unless otherwise specified, all reagents were purchased from sigma-Aldrich. BLM was sourced from Solarbio (Cat:IB0871; Beijing, China) for use in the experiments, while recombinant cytokine TGF-β1 was supplied by R&D Systems (Hong Kong, China). The primary antibody against ESRP1 (rabbit polyclonal, Cat No. 21045-1-AP), Epac (rabbit polyclonal, Cat No. 12572-1-AP), Rap1a (mouse monoclonal, Cat No. 68125-1-Ig), N-cadherin (rabbit polyclonal, Cat No. 22018-1-AP) and β-actin (rabbit polyclonal, Cat No. 20536-1-AP) were obtained from Proteintech (Wuhan, China). E-cadherin (rabbit polyclonal, Cat No.#AF0131) was purchased from Affinity (Jiangsu, China). Multiplex immunofluorescence staining was performed according to the manufacturer’s instructions using the Servicebio multiplex immunofluorescence kit (Servicebio, Wuhan, China). The istological and immunohistochemical reagents used in this study were obtained from Zhong-shan-Jin-qiao Biotechnology (Beijing, China). Quantitative real-time PCR kits were sourced from Vazyme (Nanjing, China), and siRNA along with other chemical reagents from Sangon Biotech (Shanghai, China).

### 5.3 The construction of BLM-induced mouse IPF model

The conduct of animal experiments followed the directives and approval of the Ethics Committee for Animal Research (Approval Number: IACUC-NYLAC-2023-252). Male C57BL/6J mice (22 ± 2 g, aged 6–8 weeks) were acquired from the Animal Centre of Ningxia Medical University and used to establish the IPF model. The mice were maintained in a specific pathogen-free (SPF) facility, where they had continuous access to potable water and a nutritionally balanced standard chow diet provided *ad libitum*. After intraperitoneal anesthesia with 0.1 % pentobarbital sodium (50 mg kg^−1^), IPF was established through a single intratracheal administration of bleomycin sulfate at a dose of 2 mg/kg dissolved in 50 μL of sterile 0.9% saline. Control mice received an equal volume of vehicle alone. Animals were monitored daily for body weight and general condition. On day 21 post-instillation, mice were euthanized by exsanguination under deep anesthesia, after which lungs were harvested for histopathological examination and biochemical analyses.

### 5.4 Sequencing with 10 × Genomics platform

Lung tissues from mice in the PBS and BLM groups were dissociated into single-cell suspensions and loaded onto a Chromium Single Cell Controller (10 × Genomics, United States) for single-cell library construction and sequencing. Raw base call files were processed with Cell Ranger (v6.0) to perform demultiplexing, alignment, and UMI counting, and reads were mapped to the mouse reference genome (mm10) to generate the gene–cell count matrix. Downstream analyses were conducted in R using the Seurat workflow. Cells were retained after quality control based on standard metrics (including the number of detected genes/UMIs and the proportion of mitochondrial transcripts), and low-quality cells were removed. The filtered data were normalized and scaled, highly variable genes were identified, and principal component analysis (PCA) was performed. Graph-based clustering was conducted using a shared nearest neighbor (SNN) graph built on the top 10 principal components, and visualization was generated with Uniform Manifold Approximation and Projection (UMAP; RunUMAP). Differentially expressed genes (DEGs) for each cluster (or between groups when applicable) were identified using a Wilcoxon rank-sum test, and multiple testing correction was applied to control the false discovery rate (FDR). Functional enrichment analyses were performed for GO terms and KEGG pathways using curated databases, and significantly enriched terms/pathways were defined based on adjusted *P*-values.

### 5.5 Cell culture and the construction of EMT cell model

According to the manufacturer’s guidelines, the MLE-12 were acquired from the Shanghai iCell Bioscience Institute (iCell-m036) and cultured in DMEM/F12 basal medium with added supplements (iCell-0033a). Cells were maintained in a humidified incubator under sterile conditions at 37°C with 5% CO2 until reaching 80–90% confluence. After seeding in 6-well plates overnight, the cells were stimulated with TGF-β_1_ (10 ng/mL) for 24 h to construct EMT cell model and then collected for qPCR and western blot.

### 5.6 Lentiviral transduction and siRNA transfection

MLE-12 cells were plated into 6-well plates then infected with the designated lentiviral constructs plus 2 μg/ml polybrene at 70% confluence overnight, followed by 2 μg/mL puromycin selection for 3 days. siRNA duplexes targeting gene-*epac/rap1a* were transfected at 50 nM using Lipofectamine RNAiMAX according to the manufacturer’s instructions. After 48 h, qPCR and western blot assays were employed to verify knockdown efficiency. The sequence of siRNA used in present study was attached to Table 2. The sequences of the siRNAs (*epac* and *raap1a*) used in the present study are listed in Table 3.

**TABLE 2:** Primers used in this study (Sangon Biotech).

Gene name	Gene ID	Forward primer (5’–3’)	Reverse primer (5’–3’)
Esrp1	207,920	CAAGCTGGGTTCGGATGAGAA	AGGTTTTCGGCGTCTATTTTAGT
N-cadherin	12,558	AGCGCAGTCTTACCGAAGG	TCGCTGCTTTCATACTGAACTTT
Fibronectin	14,268	ATGTGGACCCCTCCTG ATAGT	GCCCAGTGATTTCAGCAAAGG
Vimentin	22,352	TCCACACGCACCTACA GTCT	CCGAGGACCGGGTCACATA
α-SMA	11475	GTCCCAGACATCAGGG AGTAA	TCGGATACTTCAGCGTCAGGA
Zeb1	21,417	GCTGGCAAGACAACGT GAAAG	GCCTCAGGATAAATGACGGC
Epac	223,864	GCACGCTGCTCAATATGGTG	CGGTGCTCGAACACTAGCTG
Rap1a	109,905	ATGCGTGAGTACAAGCTAGTAGT	AATCTACCTCGACTTGCTTTCTG
β-actin	11,461	CATGTACGTTGCTATCCAGGC	CTCCTTAATGTCACGCACGAT
Rap1a	109,905	ATGCGTGAGTACAAGCTAGTAGT	AATCTACCTCGACTTGCTTTCTG
Epac	223,864	GCACGCTGCTCAATATGGTG	CGGTGCTCGAACACTAGCTG

**TABLE 3:** siRNA sequence used in this study (Sangon Biotech).

Gene name	Sense	AntiSense
*epac*	GCUGGACACCACUUACCAA/ dT//dT/	UUGGUAAGUGGUGUCCAGC/ dT//dT/
*rap1a*	CAGCAAUGAGGGAUUUGUA/ dT//dT/	UACAAAUCCCUCAUUGCUG/ dT//dT/

*FGFR2-IIIb* and *FGFR2-IIIc*, alternative splicing isoforms of Fibroblast Growth Factor Receptor 2.

### 5.7 Histopathology

Left lungs were fixed overnight in 4% paraformaldehyde, embedded in paraffin, and cut into 4 μm sections. After deparaffinization and rehydration, the slides were stained with hematoxylin and eosin (H&E) to examine tissue architecture and with Masson’s trichrome to evaluate collagen distribution, following the manufacturer’s recommendations. Additional sections were subjected to immunohistochemical analysis.

### 5.8 Immunohistochemistry (IHC) analysis

Cells were first dewaxing and rehydration, followed by antigen retrieval. Endogenous peroxidase activity was removed and incubated with 5% BSA. 1 h later, the sections were stained overnight at 4°C with rabbit anti-Fn (Abcam; Cat: ab2413; Dilution: 1:400), rabbit anti-Vim (Abcam; Cat: ab92547; Dilution: 1:600), rabbit anti-α-SMA (CST; Cat:19245S; Dilution: 1:200), and rabbit anti-ESRP1 (Proteintech;Cat No. 21045-1-AP; Dilution: 1:100), Epac (Proteintech; Cat No. 12572-1-AP; Dilution: 1:200), Rap1a (Proteintech; Cat No. 68125-1-Ig; Dilution: 1:50) and subsequently incubated with the streptavidin-biotin complex (ABC) detection kit (BOSTER, China) for 30 min at room temperature. Each section underwent color development using peroxidase-linked avidin/biotin and 3’-3-diaminobenzidine (DAB) substrate from ZSGB-BIO, Cat# PV-6000D, followed by haematoxylin counterstaining.

### 5.9 Immunofluorescence analysis

Paraffin-embedded sections were dewaxed, rehydrated, and subjected to antigen retrieval using 10 mM sodium citrate buffer (pH 6.0) at 95°C for 15 min. Endogenous peroxidase activity was neutralized with 3% hydrogen peroxide for 10 min, and nonspecific binding was blocked with 5% normal donkey serum for 1 h at room temperature. Slides were then incubated overnight at 4°C with the respective primary antibodies (ESRP1 1:200; Rap1a 1:200) in a humidified chamber. After three 5-min PBS washes, slides were treated with HRP-conjugated secondary antibody for 1 h and developed with iF488-TSA (10 min, RT, dark). A second round of heat-mediated antigen retrieval was performed, followed by incubation with anti-EpCAM (1:100) and the corresponding secondary antibody and iF555-TSA using the same washing regime. Nuclei were counterstained with DAPI (4’,6-Diamidino-2-Phenylindole, Dihydrochloride) for 10 min, autofluorescence quenched for 5 min and sections rinsed under running water for 10 min. Slides were mounted in Prolong Diamond and imaged with a Leica DMi8 confocal microscope.

### 5.10 Hydroxyproline assay

The concentration of hydroxyproline in the lungs was measured with a commercial ELISA (Enzyme-Linked Immunosorbent Assay) kit (A030-2-1) from Nanjing Jiancheng Bioengineering Institute, following the manufacturer’s guidelines.

### 5.11 Western blotting analysis

Whole proteins were isolated using the Whole Cell Lysis Assay kit (KeyGEN5303, Nanjing, China). Cytoplasmic and nuclear proteins were isolated using the Nuclear and Cytoplasmic Protein Extraction Kit (KGB5302, Nanjing, China) according to the manufacturer’s instructions. Cells were collected and incubated with a cytoplasmic extraction buffer on ice, followed by centrifugation to obtain the cytoplasmic supernatant. The remaining pellets were then resuspended in a nuclear extraction buffer to recover nuclear proteins. Histone-3 and β-actin were employed as internal controls for the nuclear and cytoplasmic fractions, respectively. Equal protein quantities from each sample were separated on a 10% gel, transferred to PVDF membranes, and probed with primary antibodies at 4°C overnight followed by appropriate secondary antibodies (Proteintech, SA00001-2). The protein bands were identified and measured using Superstar ECL (KeyGEN, Nanjing, China) with the Gel Imaging System (Amersham Imagequant800uv, United States). The results were quantitative analysis by using Image J. The antibodies utilized in this research include: rabbit anti-N-cad (1:1,000), rabbit anti-E-cad (1:1,000), mouse anti-Rap1a (1:1,000), rabbit anti-Epac (1:500), and rabbit anti-Esrp1 (1:1,000) and anti-β-actin (1:2,500) polyclonal antibodies were purchased from Proteintech (Wuhan, Hubei, China). The relative expression of target proteins was determined by comparing the gray level ratio of the target bands to β-actin, which served as an internal control.

### 5.12 Quantitative PCR

RNA extraction was carried out with TRIzol reagent (Ambion, United States). A total of 1 μg RNA was then subjected to reverse transcription using a cDNA synthesis kit, following the manufacturer’s instructions (Vazyme Biotech, Nanjing, China). quantitative PCR was performed on a Bio-Rad IQ5 system (Bio-Rad Inc., United States) using HiScript Q RT SuperMix for qPCR (Vazyme, Nanjing, China). Expression levels were normalized against β-actin as the housekeeping gene, and fold changes were computed by applying the 2^–△△CT^ algorithm. Details of the primer sequences designed for the amplification of mouse genes are presented in Supplementary Table 1.

### 5.13 Co-immunoprecipitation

Cells at 80% confluence were washed twice with chilled PBS and lysed for 30 min on ice using RIPA buffer supplemented with protease inhibitors. Lysates were centrifuged at 12,000 rpm for 15 min at 4°C, and the supernatant was designated as the total protein extract. A minor aliquot of the lysate was retained as the input control. Co-IP was performed with the Beyotime Co-IP kit (P2179S) according to the manufacturer’s instructions.

Briefly, the total protein extract was incubated with primary antibodies against ESRP1 (rabbit polyclonal, ProteinTech, Cat No. 21045-1-AP) or Epac (rabbit polyclonal, ProteinTech, Cat No. 12572-1-AP) at 4°C overnight with gentle rotation. For the negative control, an equal amount of normal rabbit IgG (ProteinTech, Cat No. B900610) was used instead of the specific primary antibody. Subsequently, protein A/G agarose beads (included in the Beyotime Co-IP kit) were added to the mixture and incubated for another 4 h at 4°C. After incubation, the beads were washed five times with ice-cold IP wash buffer (provided in the kit) to remove non-specific bindings, and the immunoprecipitated complexes were eluted with elution buffer (included in the kit) by boiling at 95°C for 10 min. The eluted samples, along with the input control, were subjected to Western blot analysis as described below.

### 5.14 Statistical analysis

All data in this study were derived from a minimum of three independent experiments for each condition. Statistical analysis was performed using GraphPad Prism 9.5 (GraphPad Software Inc., United States). Statistical analyses included one-way ANOVA for comparisons among multiple groups with a common control and Student’s *t*-tests for pairwise analyses. Data are expressed as mean ± SD. A probability value of < 0.05 was regarded as statistically significant, with symbols denoting significance as follows: *P < 0.05, **P < 0.01, ***P < 0.001, and ****P < 0.0001.

## Data Availability

The original contributions presented in this study are included in this article/Supplementary Material, further inquiries can be directed to the corresponding authors.
