# Supplementary figures and images for: ESRP1 drives epithelial-mesenchymal transition by activating EPAC-RAP1A signaling axis
Source: Front Med (Lausanne). 2026 Feb 6;13:1734619. doi: 10.3389/fmed.2026.1734619 (PMC12920467; doi:10.3389/fmed.2026.1734619)

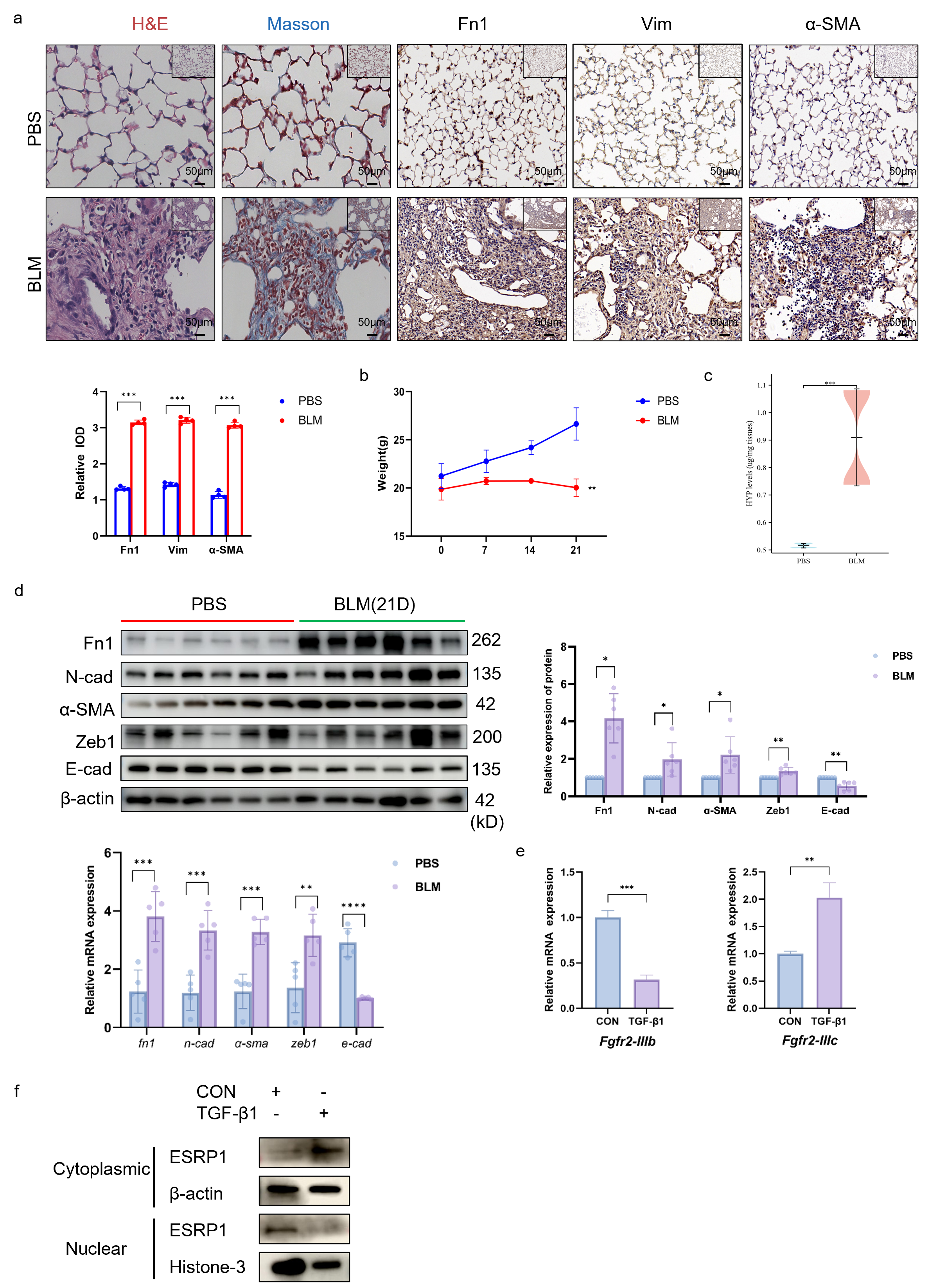

Supplement: Supplementary file 1 [file Image_1.tif]
